# Higher levels of serum α-Klotho are longitudinally associated with less central obesity in girls experiencing weight gain

**DOI:** 10.3389/fendo.2023.1218949

**Published:** 2023-07-14

**Authors:** Gemma Carreras-Badosa, Elsa Puerto-Carranza, Berta Mas-Parés, Ariadna Gómez-Vilarrubla, Bernat Gómez-Herrera, Ferran Díaz-Roldán, Elena Riera-Pérez, Francis de Zegher, Lourdes Ibañez, Judit Bassols, Abel López-Bermejo

**Affiliations:** ^1^Pediatric Endocrinology Group, Girona Biomedical Research Institute, Girona, Spain; ^2^Pediatrics, Dr. JosepTrueta Hospital, Girona, Spain; ^3^Maternal-Fetal Metabolic Group, Girona Biomedical Research Institute, Girona, Spain; ^4^Pediatrics, Fundació Salut Empordà, Figueres, Spain; ^5^Department of Development & Regeneration, University of Leuven, Leuven, Belgium; ^6^Sant Joan de Déu Children’s Hospital Pediatric Research Institute, University of Barcelona, Barcelona, Spain; ^7^CIBER de Diabetes y Enfermedades Metabólicas Asociadas, Instituto de Salud Carlos III, Madrid, Spain; ^8^Department of Medical Sciences, University of Girona, Girona, Spain

**Keywords:** Klotho, central obesity, weight gain, visceral fat, pediatrics

## Abstract

**Introduction:**

Klotho is an anti-aging protein that reduces adiposity and increases caloric expenditure, among others. Although associations between secreted α-Klotho levels and obesity have been described, its relationship with central obesity and visceral fat accumulation during childhood is poorly understood. Our objective was to study the longitudinal associations between serum α-Klotho concentrations and obesity-related parameters in apparently healthy children.

**Subjects and methods:**

We studied a cohort of 208 apparently healthy school-age children (107 girls and 101 boys) assessed at baseline (mean age 8.5 ± 1.8 years) and at follow-up 4 years later. Serum α-Klotho concentrations were measured at baseline in all subjects. Obesity-related parameters, such as BMI, waist circumference, body fat, visceral fat, triglyceride levels, HOMA-IR index, and C-reactive protein were studied. Boys and girls were classified into 3 groups according to weight change between baseline and follow-up visits: weight loss, stable weight, or weight gain (based on a BMI-SDS change cut-off > 0.35 SD).

**Results:**

In girls (N=107), but not in boys, we observed negative associations of serum α-Klotho protein with BMI, waist circumference, body fat, visceral fat, HOMA IR index, and C-reactive protein at baseline and also at follow-up. The associations of α-Klotho and obesity-related parameters were more evident in girls who exhibited weight gain. In such girls, multivariate regression analyses (adjusting for age, puberty and baseline weight/height ratio) showed that α-Klotho protein was negatively associated with follow-up BMI, waist circumference, and visceral fat (p = 0.003 to 0.028). For each 1 SD-increase in baseline α-Klotho, follow-up waist circumference decreased by 4.15 cm and visceral fat by 1.38 mm.

**Conclusions:**

In school-age girls, serum α-Klotho concentrations are longitudinally related to a more favorable metabolic profile. In girls experiencing weight gain, α-Klotho may prove to be a protective factor against the accumulation of visceral fat.

## 1 Introduction

Klotho is a transmembrane protein widely known for its anti-aging effects (1) as well as for its protective effect on the prevention of cardiovascular diseases (2). Although Klotho is mainly expressed in the kidneys with a major role in ion homeostasis and mineral metabolism (3), it is also known to be expressed in adipose tissue with implications in insulin signaling, adipogenesis and glucose metabolism (4).

Klotho has been related to caloric expenditure (5) and to reduced adiposity in adults (6) as well as in children (7). Specifically, studies have associated reduced α-Klotho with higher abdominal obesity in middle-aged and older women (8). In this study, women who developed obesity during their lifetime had consistently lower klotho levels than their never-obese counterparts (8). Others have shown increased α-Klotho levels to be associated with a reduction of visceral fat after a moderate intensity endurance training in adult men (9).

Recently, klotho has gained broad attention in female reproductive diseases, with various physiological and pathological role of Klotho in the hypothalamus-pituitary-ovary axis. In women, klotho plays a vital role in regulating the secretion of GnRH and is also needed to sustain normal ovary function. Hence, klotho is involved in female central hypogonadotropic hypogonadism, polycystic ovary syndrome and primary ovarian failure (10).

Despite these recent studies, little is known about the association between α-Klotho and central obesity or visceral fat accumulation in children.

We hypothesized that higher levels of circulating α-Klotho would be associated with less central obesity in apparently healthy children. Therefore, our objective was to study the longitudinal associations between serum α-Klotho and several obesity-related parameters in a population of apparently healthy children at baseline and also at follow-up (after 4 years), and to study whether sex or weight gain could modulate any potential associations.

## 2 Methods

A study population of 208 apparently healthy school-age children (107 girls and 101 boys) were consecutively recruited among those seen in primary care settings in Girona, a region in North-eastern Spain, as previously reported (11). A follow-up visit was offered to all the families after 4 years of the initial baseline visit, and longitudinal follow-up data was available in all the studied children. The study protocol was approved by the Ethics Review Committee of the Institutional Review Board of Dr Josep Trueta Hospital and was performed in accordance with their code of ethics, guidelines and regulations. Informed written consent was obtained from the parents. Briefly, inclusion criteria were age between 6 and 10 years. Exclusion criteria were (i) major congenital anomalies; (ii) abnormal blood count, abnormal liver, kidney or thyroid functions; (iii) evidence of chronic illness or prolonged use of medication; (iv) acute illness or use of medication in the month preceding potential enrolment.

Clinical examination followed by venous blood sampling in the fasting state was performed in the morning as previously reported (11). Briefly, weight and height were measured with a calibrated scale and a Harpenden stadiometer, respectively. BMI was calculated as weight divided by the square of height in meters. Age-adjusted and sex-adjusted standard deviation scores (SDS) for BMI were calculated using regional normative data (12). Waist circumference was measured in the supine position at the umbilical level and hip circumference was measured at the widest part, at the level of the greater trochanters, with a metric tape. Body surface area (BSA) was calculated using Mosteller’s formula: (√weight*height)/60 (13). Body composition was assessed by bioelectric impedance (Hydra Bioimpedance Analyzer 4200, Xitron Technologies, San Diego, CA). Fat mass (FM) percentage was calculated using the body weight and lean mass parameters [FM = (body weight – lean mass)/*100]. Puberty was evaluated according to Tanner criteria. Visceral fat was estimated by ultrasonography (MyLab 25; Esaote, Italy) and calculated as described by Hirooka et al. (14) using the distance between the internal surface of the abdominal wall and the posterior wall of the aorta at the umbilical level and the thickness of the fat layer of the posterior right renal wall in the perinephric space. Visceral fat measured by ultrasound has been shown to correlate well with that measured by computed tomography (14). Images were obtained in the supine position at the end of a normal exhalation, using a convex 3.5-MHz transducer. Averages of three measurements of each parameter were used in the study. The same observer, who was unaware of the clinical and laboratory characteristics of the subjects, performed all ultrasound measurements. Intra-subject coefficient of variation for ultrasound measurements was less than 6%. All serum samples were obtained between 8:00 and 9:00 AM under fasting conditions. Fasting serum immunoreactive glucose was assayed by the hexokinase method (Cobas C, Roche Diagnostics, Indianapolis, USA) and insulin was measured by immunochemiluminiscence (IMMULITE 2000, Diagnostic Products, Los Angeles, CA, USA). The lower detection limit was 2.0 mg/dL 0.4 mIU/L and intra- and inter-assay CVs were < 3% and < 10%, respectively. Insulin resistance was estimated by the homeostasis model assessment index: HOMA-IR = (fasting insulin in mU/L * fasting glucose in mg/dL)/405. Total serum triacylglycerol (triglycerides) was measured by glycerol-phosphate oxidase method (ARCHITECT, Abbott Laboratories, Abbott Park, IL, USA). Lower detection limit was 5.0 mg/dL, and intra- and inter-assay CVs < 5%. Serum levels of high-sensitivity C-reactive protein (hsCRP) were measured using the ultrasensitive latex immunoassay CRP Vario (Sentinel Diagnostics, Milan, Italy). Lower detection limit was 0.1 mg/l, and the intra- and interassay CVs were <3%. α-Klotho was measured using the Human soluble α-Klotho assay kit (Immuno-Biological Laboratories Co. IBL, Ltd., Japan), with a measurement range of 93.75-6000 pg/mL, sensitivity of 6.15 pg/mL and intra- and inter-assay CV value of 3.1% and 6.9% respectively.

Follow-up data was obtained 4 years after the baseline visit and the same anthropometric, and clinical and laboratory variables were assessed following the same methodology. BMI-SDS change between baseline and follow-up visits was calculated and the studied children were classified into 3 groups: those experiencing weight loss, those having a stable weight, and those experiencing weight gain. The cut-off value chosen to define these groups was set at BMI-SDS change > 0.35 SD (which corresponds approximately to half the change in BMI defined as rapid weight change (15). This weight change cutoff value also provided fairly balanced groups of subjects in our studied cohort).

Statistical analyses were performed using SPSS version 22.0 (SPSS Inc.). Results are expressed as mean ± standard deviation (SD) for the continuous variables or percentage (%) for categorical variables. Logarithmic transformation was used to obtain normally distributed values for triglycerides and α-Klotho. Differences across weight change subgroups defined by BMI-SDS change tertiles were examined by one-way ANOVA test (continuous data) and by Chi-square (categorical data). The ANOVA results were corrected for multiple comparisons using the Bonferroni *post-hoc* test. Levels of α-Klotho were correlated with anthropometric and clinical variables using Pearson bivariate correlations. Correlation analyses were followed by multivariate linear regression analyses in which the enter method was used for computing the independent variables (multivariate analyses were adjusted for covariates: sex, baseline age and baseline BSA or weight-to-height ratio) and the step wise method was used for computing effect size of the observed associations. The significance level was set at p ≤ 0.05.

## 3 Results

### 3.1 Associations between baseline α-klotho and obesity-related parametres

In the subgroup of children who experienced weight gain, baseline α-klotho levels were associated inversely and independently with baseline BMI, waist, waist-to-height ratio, visceral fat, triglycerides and hsCRP and longitudinally with follow-up weight, BMI, waist, waist-to-height ratio, body fat mass and visceral fat (all p <0.05, **Table 1**). Most of these associations were independent from sex, baseline age, puberty and either baseline BSA or baseline weight-for-height (as proxies for BMI) in multivariate analyses (**Table 1**).

**Table 1:** Correlations between baseline α-Klotho and the obesity-related parameters studied in all population and according to subgroups of weight change between baseline and follow-up.

	All population (N=208)	Weight loss (N=59)	Stable weight (N=76)	Weight gain (N=74)
Baseline	Baseline α-Klotho (log)	Baseline α-Klotho (log)	Baseline α-Klotho (log)	Baseline α-Klotho (log)
BMI	-0,310***	-0,419**	-0,183	-0,362** †
BMI SDS	-0,272***	-0,368**	-0,118	-0,357**
Waist	-0,301***	-0,334* †	-0,215	-0,369**
Waist-to-height ratio	-0,250***	-0,216	-0,148	-0,365**
Body fat mass	-0,304***	-0,457**	-0,294*	-0,244
Visceral fat	-0,313***	-0,234	-0,291*	-0,395**
Triglycerides (log)	-0,177*	-0,216	-0,065	-0,262* †
HOMA-IR	-0,212**	-0,405**	-0,179	-0,130
hsCRP (log)	-0,207**	-0,219	-0,032	-0,346** †
**Follow-up**	Baseline α-Klotho (log)	Baseline α-Klotho (log)	Baseline α-Klotho (log)	Baseline α-Klotho (log)
BMI	-0,237**	-0,263*	-0,170	-0,402** †
BMI SDS	-0,193**	-0,223	-0,114	-0,345**
Waist	-0,284***	-0,358**	-0,207	-0,427*** †
Waist-to-height ratio	-0,215**	-0,194	-0,146	-0,400** †
Body fat mass	-0,107	-0,036	-0,032	-0,278*
Visceral fat	-0,272***	-0,252	-0,195	-0,473*** †
Triglycerides (log)	-0,113	-0,252	-0,074	-0,126
HOMA-IR	-0,142*	-0,225	-0,081	-0,191
hsCRP (log)	-0,151*	-0,193	-0,172	-0,167

Pearson correlation coefficients (r) are shown; *p value <0.05, ** p value <0.01, and ***p value <0.001.

Significant independent associations after correcting for sex, baseline age, baseline puberty status and baseline body surface area (BSA) are highlighted **in bold**; and significant independent associations after correcting for sex, baseline age, baseline puberty status and baseline weight-for-height ratio are highlighted with †.

At baseline, the subgroup of children who had a stable weight had lower values for most of the obesity parameters, as compared to the other subgroups; (all p<0.05, **Table 2**), whereas at follow-up, the subgroup of children who experienced weight gain showed higher values for the obesity parameters, as compared to other subgroups, (all p<0.05, **Table 2**). Indeed, at follow up, there were no differences in the studied parametres between children who had lost weight and those who had maintained a stable weight.

**Table 2:** Descriptive values of the studied parameters in all population and according to subgroups of weight change between baseline and follow-up.

	All population (N=208)	Weight loss (N=59)	Stable weight (N=76)	Weight gain (N=74)
Baseline	Mean ± SD	Mean ± SD	Mean ± SD	Mean ± SD
Sex (female %)	51.4	45.8	52.6	54.8
Age (years)	8.5 ± 1.8	8.3 ± 1.8	8.3 ± 1.7	8.8 ± 1.8
Puberty (M/G Tanner ≥ 2; %)	14.4	13.6	**6.6 ^#^**	23.3
Obesity (BMI-SDS > 2; %)	20.2	25.4	11.8	24.7
Weight (kg)	37.8 ± 14.7	39.6 ± 14.3	**33.1 ± 12.9** ^	41.3 ± 15.7
Weight-SDS (z-score)	0.85 ± 1.47	1.29 ± 1.39	**0.36 ± 1.45** ^	1.00 ± 1.42
Height (cm)	135.1 ± 12.9	135.2 ± 12.5	132.2 ± 12.5	138.2 ± 13.1
Height-SDS (z-score)	0.68 ± 1.14	0.91 ± 1.08	0.38 ± 1.21	0.81 ± 1.04
BMI (kg/m2)	19.95 ± 4.73	20.98 ± 4.26	**18.36 ± 4.33** ^	20.77 ± 5.09
BMI-SDS (z-score)	0.65 ± 1.42	1.08 ± 1.30	**0.20 ± 1.39** ^	0.77 ± 1.44
Weight-for-height (kg/m)	0.27 ± 0.08	0.28 ± 0.08	**0.24 ± 0.07** ^	0.29 ± 0.09
Body surface area (BSA) (m^2^)	1.18 ± 0.28	1.21 ± 0.27	**1.09 ± 0.24** ^	1.24 ± 0.29
Waist (cm)	66.8 ± 13.4	69.2 ± 12.9	**62.6 ± 12.2** ^	69.1 ± 14.1
Waist-to-height ratio	0.49 ± 0.07	0.51 ± 0.07	**0.47 ± 0.07** ^	0.50 ± 0.07
Body fat mass (kg)	12.5 ± 8.2	13.7 ± 8.0	**10.0 ± 6.9 ^**	13.9 ± 9.0
Visceral fat (mm)	6.63 ± 2.31	6.86 ± 2.23	**5.99 ± 2.20 ^**	7.10 ± 2.36
Triglycerides (mg/dL)	61 ± 35	68 ± 44	**51 ± 25** ^	65 ± 33
Glucose (mg/dL)	86 ± 7	88 ± 6	85 ± 8	87 ± 6
Insulin (mcU/mL)	5.8 ± 5.7	6.1 ± 5.9	**3.8 ± 3.7** ^	6.9 ± 6.7
HOMA-IR	1.20 ± 1.04	1.33 ± 1.07	**0.81 ± 0.72** ^	1.50 ± 1.43
hsCRP (mg/L)	2.10 ± 2.03	2.46 ± 2.25	1.52 ± 1.05	2.42 ± 2.31
α-Klotho (pg/mL)	2286 ± 987	2202 ± 890	2284 ± 898	2357 ± 1144
**Follow-up**	Mean ± SD	Mean ± SD	Mean ± SD	Mean ± SD
Age (years)	12.9 ± 1.9	12.7 ± 1.9	12.6 ± 1.9	13.3 ± 1.9
Puberty (M/G Tanner ≥ 2; %)	68.8	66.7	60.0	**80.0 ^#^**
Obesity (BMI-SDS > 2; %)	21.6	3.4	15.8	**42.5 ^#^**
Weight (kg)	59.0 ± 20.0	54.8 ± 16.1	52.4 ± 18.0	69.4 ± 21.2 *
Weight-SDS	0.85 ± 1.52	0.46 ± 1.05	0.34 ± 1.39	1.72 ± 1.62 *
Height (cm)	158.3 ± 12.2	159.2 ± 12.5	155.8 ± 12.8	160.2 ± 11.2
Height-SDS	0.42 ± 1.04	0.62 ± 1.00	0.25 ± 1.15	0.44 ± 0.93
BMI (kg/m2)	23.19 ± 6.05	21.34 ± 4.32	21.14 ± 5.14	26.87 ± 6.49 *
BMI-SDS	0.77 ± 1.59	0.24 ± 1.08	0.25 ± 1.39	1.74 ± 1.70 *
Weight-for-height (kg/m)	0.36 ± 0.11	0.34 ± 0.08	0.33 ± 0.09	0.43 ± 0.11 *
Body surface area (BSA) (m^2^)	1.59 ± 0.32	1.54 ± 0.28	1.49 ± 0.30	1.74 ± 0.32 *
Waist (cm)	77.0 ± 15.2	73.1 ± 12.0	71.7 ± 12.7	86.0 ± 16.0 *
Waist-to-height ratio	0.48 ± 0.08	0.46 ± 0.07	0.46 ± 0.07	0.53 ± 0.08 *
Body fat mass (kg)	26.5 ± 8.9	23.6 ± 7.3	24.1 ± 7.8	31.3 ± 9.3 *
Visceral fat (mm)	7.20 ± 2.10	6.42 ± 1.45	6.66 ± 1.83	8.38 ± 2.31 *
Triglycerides (mg/dL)	69 ± 36	60 ± 25	67 ± 46	78 ± 29 *
Glucose (mg/dL)	86 ± 7	86 ± 6	86 ± 7	86 ± 8
Insulin (mcU/mL)	11.4 ± 6.8	9.5 ± 5.4	9.9 ± 5.1	14.4 ± 8.3 *
HOMA-IR	2.44 ± 1.53	2.06 ± 1.26	2.13 ± 1.16	3.07 ± 1.84 *
hsCRP (mg/L)	1.26 ± 1.09	0.89 ± 0.57	0.99 ± 0.47	1.83 ± 1.63 *

Data are shown as mean ± standard deviation (SD).

^#^Significant Chi-square test, p value <0.05.

*Significant post-hoc tests for ANOVA between weight gain group versus other groups, p value <0.05.

^Significant post-hoc tests for ANOVA between stable weight group versus other groups, p value <0.05.

### 3.2 Associations between baseline α-klotho and obesity-related parametres in girls

**Figure 1:**
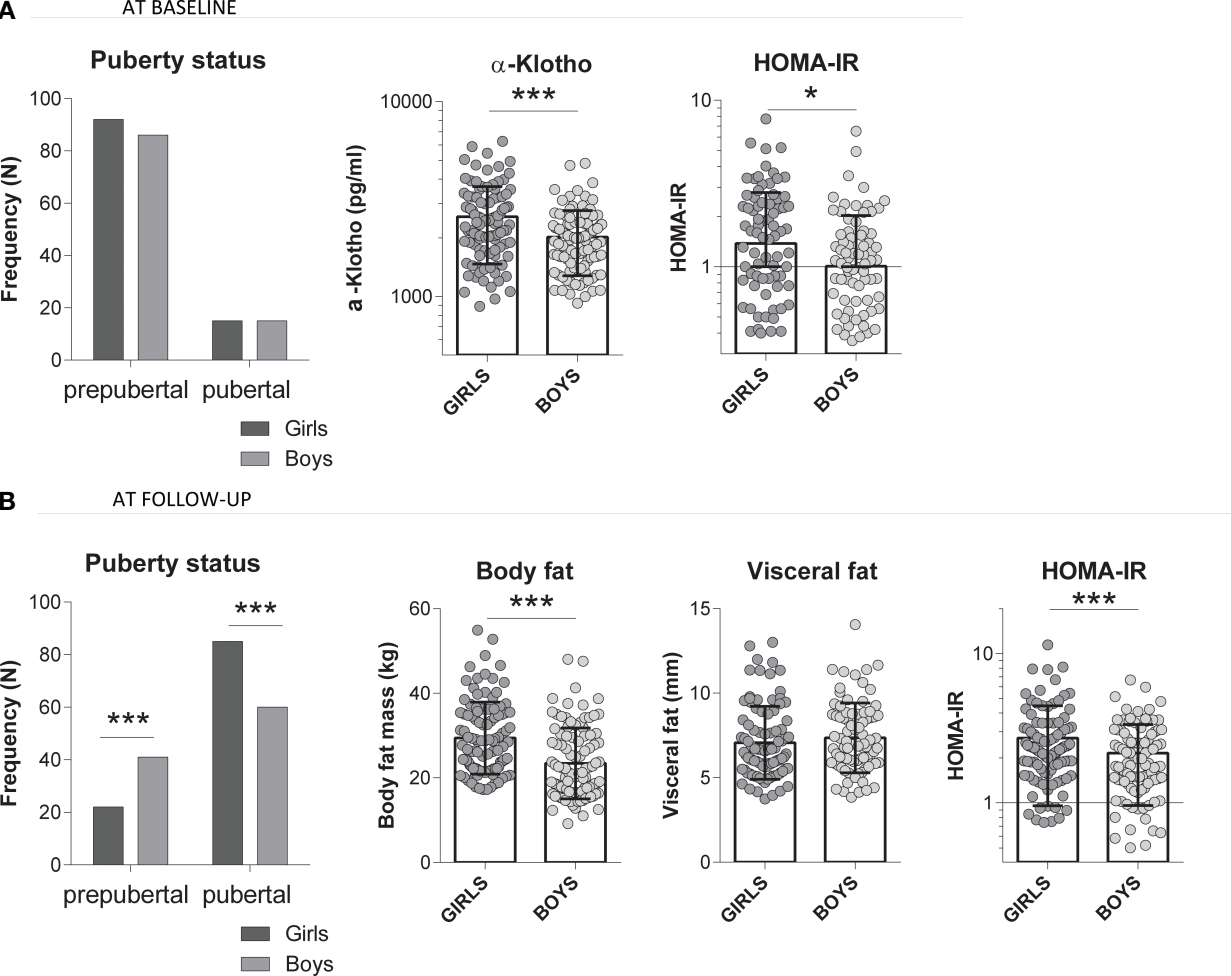
Comparison of several studied parameters in girls (N=107) and boys (N=101) at **(A)** baseline (serum α-Klotho levels, baseline HOMA-IR) and at **(B)** follow-up (total body fat mass, visceral fat and HOMA-IR). T-test comparing all girls *versus* boys, *p value <0.05, ***p value <0.001.

At baseline, girls presented higher values of triglycerides, HOMA-IR and α-Klotho as compared to boys (all p<0.05, **Table 3**). At follow-up, girls showed higher values for body fat, insulin and HOMA-IR; as well as higher percentage of pubertal subjects (all p<0.05, **Table 3**) as compared to boys. Of note, higher baseline α-Klotho concentrations in girls as compared to boys were observed in parallel to higher baseline HOMA-IR, higher follow-up body fat mass and HOMA-IR, but not with higher follow-up visceral fat (**Figure 1**).

**Table 3:** Descriptives values of the studied parameters according to sex subgroups: girls *versus* boys.

	Girls (N=107)	Boys (N=101)
Baseline	Mean ± SD	Mean ± SD
Age (years)	8.4 ± 1.8	8.6 ± 1.8
Puberty (M/G Tanner ≥ 2; %)	14.0	14.9
Obesity (BMI-SDS > 2; %)	20.6	19.8
Weight (kg)	36.9 ± 14.7	38.7 ± 14.8
Weight-SDS	0.79 ± 1.39	0.91 ± 1.55
Height (cm)	133.8 ± 13.2	136.6 ± 12.6
Height-SDS	0.60 ± 1.11	0.77 ± 1.16
BMI (kg/m2)	19.86 ± 4.75	20.05 ± 4.73
BMI-SDS	0.63 ± 1.37	0.67 ± 1.48
Weight-for-height (kg/m)	0.27 ± 0.08	0.28 ± 0.08
Body surface area (BSA) (m^2^)	1.16 ± 0.28	1.20 ± 0.28
Waist (cm)	65.7 ± 13.0	67.9 ± 13.7
Waist-to-height ratio	0.49 ± 0.07	0.49 ± 0.08
Body fat mass (kg)	13.1 ± 8.5	11.9 ± 7.9
Visceral fat (mm)	6.58 ± 2.54	6.68 ± 2.08
Triglycerides (mg/dL)	**66 ± 34 ^**	55 ± 35
Glucose (mg/dL)	87 ± 7	86 ± 7
Insulin (mcU/mL)	6.3 ± 5.9	4.6 ± 3.8
HOMA-IR	**1.38 ± 1.12 ^**	1.01 ± 0.98
hsCRP (mg/L)	2.36 ± 2.05	1.84 ± 1.71
α-Klotho (pg/mL)	**2541 ± 1118 ^**	2016 ± 740
**Follow-up**	Mean ± SD	Mean ± SD
Age (years)	12.8 ± 1.9	13.0 ± 1.9
Puberty (M/G Tanner ≥ 2; %)	78.8 *	58.2
Obesity (BMI-SDS > 2; %)	24.3	18.8
Weight (kg)	57.9 ± 19.5	60.2 ± 20.7
Weight-SDS	0.96 ± 1.58	0.76 ± 1.47
Height (cm)	**156.5 ± 10.7 ^**	160.2 ± 13.5
Height-SDS	0.45 ± 1.04	0.40 ± 1.04
BMI (kg/m2)	23.46 ± 6.46	22.91 ± 5.62
BMI-SDS	0.88 ± 1.68	0.65 ± 1.49
Weight-for-height (kg/m)	0.36 ± 0.11	0.37 ± 0.11
Body surface area (BSA) (m^2^)	1.56 ± 0.30	1.62 ± 0.34
Waist (cm)	76.7 ± 15.3	77.5 ± 15.2
Waist-to-height ratio	0.49 ± 0.08	0.48 ± 0.08
Body fat mass (kg)	**29.4 ± 8.5 ^**	23.4 ± 8.4
Visceral fat (mm)	7.06 ± 2.15	7.35 ± 2.05
Triglycerides (mg/dL)	70 ± 34	67 ± 37
Glucose (mg/dL)	86 ± 6	87 ± 8
Insulin (mcU/mL)	**12.8 ± 7.9 ^**	9.9 ± 5.1
HOMA-IR	**2.71 ± 1.75 ^**	2.15 ± 1.19
hsCRP (mg/L)	1.52 ± 0.78	0.99 ± 0.73

Data are shown as mean ± standard deviation (SD).

*Chi-squared test, p value < 0.05; ^T-test comparing girls versus boys, p value < 0.05.

We also assessed associations between baseline α-klotho and obesity-related parameters separately in girls and boys.

**Figure 2:**
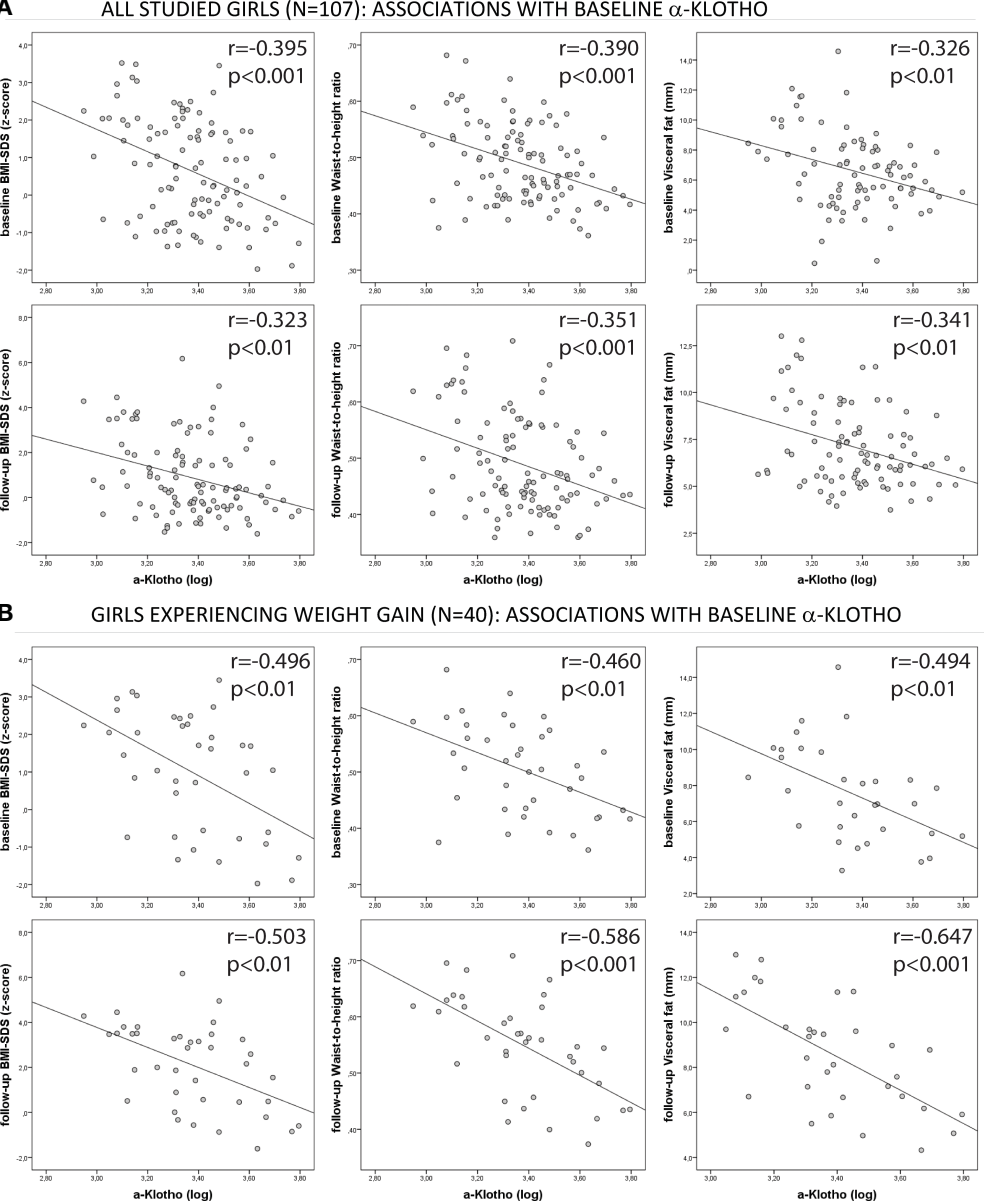
Correlation graphs between α-Klotho and the studied parameters: BMI, waist-to-height ratio and visceral fat in: **(A)** all studied girls (N=107) and **(B)** girls experiencing weight gain (N=40).

In the studied girls, and especially in those girls who experienced weight gain, α-Klotho showed inverse and independent longitudinal associations with weight, BMI, waist, waist-to-height ratio, body fat and visceral fat (all p<0.05, **Table 4**) at baseline and at follow-up after adjusting for baseline age, puberty and either baseline BSA or baseline weight-for-height (**Table 4**). The associations between α-Klotho and BMI, waist-to-height ratio, and visceral fat in all girls and in girls experiencing weight gain are shown in **Figure 2**. The effect size of the observed associations for the outcome variables related to central obesity and/or visceral fat were as follows: per each 1 SD-increase in baseline α-Klotho, follow-up waist decreased by 4.15 cm ± 1.38 (p=0.003) and follow-up visceral fat decreased by 1.38 mm ± 0.31 (p=0.003; **Table 5**).

**Table 4:** Correlations between baseline α-Klotho and the obesity-related parameters studied in all girls and according to subgroups of weight change between baseline and follow-up.

	Girls (N=107)	Weight loss (N=27)	Stable weight (N=40)	Weight gain (N=40)
Baseline	Baseline α-Klotho (log)	Baseline α-Klotho (log)	Baseline α-Klotho (log)	Baseline α-Klotho (log)
BMI	-0,387***	-0,474*	-0,276	-0,468**
BMI SDS	-0,395***	-0,509**	-0,226	-0,496**
Waist	-0,386***	-0,416*	-0,330*	-0,448**
Waist-to-height ratio	-0,390***	-0,394*	-0,306	-0,460**
Body fat mass	-0,354**	-0,474*	-0,411*	-0,362*
Visceral fat	-0,326**	-0,222	-0,378*	-0,494**
Triglycerides (log)	-0,286**	-0,267	-0,169	-0,410**
HOMA-IR	-0,245*	-0,345	-0,222	-0,271
hsCRP (log)	-0,327**	-0,387	-0,096	-0,503**
**Follow-up**	Baseline α-Klotho (log)	Baseline α-Klotho (log)	Baseline α-Klotho (log)	Baseline α-Klotho (log)
BMI	-0,347***	-0,355	-0,244	-0,564*** †
BMI SDS	-0,323**	-0,365	-0,220	-0,503**
Waist	-0,381***	-0,404*	-0,323*	-0,569*** †
Waist-to-height ratio	-0,351***	-0,317	-0,255	-0,586*** †
Body fat mass	-0,357***	-0,417*	-0,274	-0,507**
Visceral fat	-0,341**	-0,422*	-0,110	-0,647*** †
Triglycerides (log)	-0,136	-0,257	-0,044	-0,251
HOMA-IR	-0,222*	-0,279	-0,146	-0,293
hsCRP (log)	-0,236*	-0,312	-0,286	-0,232

Pearson correlation coefficients (r) are shown; *p value <0.05, **p value <0.01, and ***p value <0.001.

Significant independent associations after correcting for baseline age, baseline puberty status and baseline body surface area (BSA) are highlighted in bold; and significant independent associations after correcting for baseline age, baseline puberty status and baseline weight-for-height ratio are highlighted with †.

**Table 5:** Effect size per 1-SD-increase in baseline α-Klotho (pg/mL) in girls experiencing weight gain.

GIRLS EXPERIENCING WEIGHT GAIN (N=40)	Independent variable: 1 SD-higher baseline α-Klotho (pg/mL)	
Follow-up dependent variables:	*B value*	*SE value*
Waist (cm)	- 4.15 **	1.38
Visceral fat (mm)	- 1.38 **	0.31

Unadjusted multivariate regression coefficient (B) and SE are shown; **p value <0.01.

These results remained essentially unchanged if pubertal subjects were excluded from the study or if different cutoff points were used to define weight change (such as BMI-SDS change > 0.5 SD), but statistical power decreased as a result of smaller sample sizes (data not shown).

Finally, in the studied boys, α-Klotho did not show any independent association either at baseline or at follow-up (**Supplementary Table S1**).

## 4 Discussion

We describe several longitudinal associations between α-Klotho and obesity-related parameters, such as BMI, waist circumference, body fat, visceral fat and HOMA-IR in apparently healthy children, and highlight the inverse and longitudinal association between α-Klotho and markers of central obesity in girls experiencing weight gain.

Klotho is a transmembrane protein that acts as a co-receptor for the adipokines of the fibroblast growth factor (FGF) family (16), which regulate adipocyte development and metabolism. Multiple studies linked FGF signaling and weight change (17–19), but studies relating Klotho and central obesity in childhood are scarce. High Klotho levels have been reported in adolescents with obesity (20). Recent studies focus also on the anti-obesogenic and anti-diabetic effects of Klotho (5).

Klotho has been found to exert anti-obesogenic effects via peripheral mechanisms, such as increased lipid oxidation in liver and adipose tissue and increased whole-body energy expenditure (5). Along this line, reduced β-Klotho levels (a protein member of the Klotho subfamily) have been reported in visceral adipose tissue from adult patients with obesity (21). Studies using animal models showed that α-Klotho administration decreased lipogenesis and reduced liver lipid accumulation (22). Others have linked α-Klotho as a mediator of the central action on the whole-body energy balance and pathophysiology of obesity (23, 24). These reports on the anti-obesogenic effects of Klotho are in line with our observation of α-Klotho being related to less central obesity in school aged children, especially in girls.

Sex differences in circulating Klotho exist and have been linked to life expectancy in many species (25). Higher α-Klotho levels in humans are associated with longer life expectancy (26), and lower rates of morbidity factors such as cardiovascular disease (27). In agreement with our results, several authors have previously described that girls and women have higher levels of Klotho than boys and men of the same age (28–30); however, the mechanism explaining this sex difference is not known. Some indicated that Klotho turnover in women is not limited to the classical estrogen regulation (31), but higher Klotho levels in girls may result from a feedback mechanism due to higher vitamin D and/or IFG-1 in school-age girls (30).

Moreover, recent attention has been given to Klotho as being closely related to the hypothalamus-pituitary-ovary axis (10). Such observations indicate a role of Klotho in the regulation of the female reproductive and endocrine systems. The role of Klotho in the female endocrine system could be an additional explanations of the observed sex-dimorphic association of Klotho in relation to central obesity in our study. It is worth mentioning that despite the fact that girls in our study showed higher values of body fat, as compared to boys, the same girls did not show any differences in visceral fat as compared to boys. This observation, jointly with the observed higher concentrations of α-Klotho in girls, could indicate a protective role of α-Klotho, among others, against the accumulation of visceral fat, especially in girls. It is plausible to speculate that Klotho could contribute to the sexual dimorphism in fat accumulation patterns, as females accrue less visceral fat, especially in late adult life, which affords them protection from cardio-metabolic risk as compared to males (32, 33).

The main strength of our study is its longitudinal design. As for limitations, the analyses of subgroups of children (girls versus boys and girls with weight gain vs. other groups) limited in part the statistical power of the initial set of subjects, and thus our results need further confirmation in additional studies. BMI was highly correlated with α-Klotho (p<0.0001) in our cohort and therefore we chose to use alternative measures of body mass (such as BSA and weight-to-height ratio (34)) to analyze the independent association between α-Klotho and central obesity parameters. Finally, α-Klotho measurements were not available at follow-up in the present study.

Another limitation is the lack of physical activity data in our subjects, as recent studies have described Klotho as an emerging exerkine (35) and as a factor with ergogenic potential, which can enhance physical performance (36). Klotho levels increase with exercise (9, 37), regardless of the health condition of the individual (35) or the physical fitness (38) and this may explain, at least in part, the inverse association between physical fitness and cardiometabolic risk in school-age children (39, 40).

In conclusion, serum α-Klotho concentrations are longitudinally associated with a more favorable metabolic profile and inversely associated with central obesity parameters in school-aged children. α-Klotho protein could be a potential protective factor against the accumulation of visceral fat, especially in girls experiencing weight gain.

## Data availability statement

The raw data supporting the conclusions of this article will be made available by the authors, without undue reservation.

## Ethics statement

The studies involving human participants were reviewed and approved by Ethics Review Committee of the Institutional Review Board of Dr. Josep Trueta Hospital. Written informed consent to participate in this study was provided by the participants’ legal guardian/next of kin.

## Author contributions

GC-B and EP-C performed data analysis and wrote the first draft of the manuscript. BM-P carried out experiments and reviewed the manuscript. AG-V, BG-H, FD-R, and ER-P contributed to data collection and reviewed the manuscript. FdZ, LI, and JB reviewed the manuscript. AL-B conceived the study and contributed to writing the manuscript. All authors contributed to the article and approved the submitted version.

## References

[1] Abraham CR Li A. Aging-suppressor klotho: prospects in diagnostics and therapeutics. Ageing Res Rev (2022) 82. doi: 10.1016/j.arr.2022.101766 36283617

[2] Sun X Chen L He Y Zheng L. Circulating α-klotho levels in relation to cardiovascular diseases: a mendelian randomization study. Front Endocrinol (Lausanne) (2022) 13. doi: 10.3389/fendo.2022.842846 PMC8859151 35197934

[3] Kim J-H Hwang K-H Park K-S Kong ID Cha S-K. Biological role of anti-aging protein klotho. J lifestyle Med (2015) 5(1):1–6. doi: 10.15280/jlm.2015.5.1.1 26528423 PMC4608225

[4] Ohnishi M Kato S Akiyoshi J Atfi A Razzaque MS. Dietary and genetic evidence for enhancing glucose metabolism and reducing obesity by inhibiting klotho functions. FASEB J (2011) 25(6):2031–9. doi: 10.1096/fj.10-167056 PMC3101030 21382979

[5] Landry T Shookster D Huang H. Circulating α-klotho regulates metabolism via distinct central and peripheral mechanisms. Metabolism (2021) 121. doi: 10.1016/j.metabol.2021.154819 PMC8277751 34153302

[6] Amitani M Asakawa A Amitani H Kaimoto K Sameshima N Koyama KI. Plasma klotho levels decrease in both anorexia nervosa and obesity. Nutrition (2013) 29(9):1106–9. doi: 10.1016/j.nut.2013.02.005 23790542

[7] Kutluturk Y Akinci A Ozerol IH Yologlu S. The relationship between serum FGF-23 concentration and insulin resistance, prediabetes and dyslipidemia in obese children and adolescents. J Pediatr Endocrinol Metab (2019) 32(7):707–14. doi: 10.1515/jpem-2018-0507 31211688

[8] Orces CH. The association of obesity and the antiaging humoral factor klotho in middle-aged and older adults. Soto-Blanco B editor ScientificWorldJournal (2022) 2022:1–6. doi: 10.1155/2022/7274858 PMC9433301 36061981

[9] Middelbeek RJW Motiani P Brandt N Nigro P Zheng J Virtanen KA. Exercise intensity regulates cytokine and klotho responses in men. Nutr Diabetes (2021) 11(1). doi: 10.1038/s41387-020-00144-x PMC7791135 33414377

[10] Xie T Ye W Liu J Zhou L Song Y. The emerging key role of klotho in the hypothalamus-Pituitary-Ovarian axis. Reprod Sci (2021) 28(2). doi: 10.1007/s43032-020-00277-5 32783104

[11] Xargay-Torrent S Puerto-Carranza E Marcelo I Mas-Parés B Gómez-Vilarrubla A Martínez-Calcerrada J. Estimated glomerular filtration rate and cardiometabolic risk factors in a longitudinal cohort of children. Sci Rep (2021) 11(1). doi: 10.1038/s41598-021-91162-x PMC8175594 34083639

[12] Carrascosa Lezcano A Fernández García JM Fernández Ramos C Ferrández Longás A López-Siguero JP Sánchez González E. [Spanish cross-sectional growth study 2008. part II. height, weight and body mass index values from birth to adulthood]. Pediatr (2008) 68(6):552–69. doi: 10.1157/13123287 18559194

[13] RD M. Simplified calculation of body-surface area. N Engl J Med (1987) 317(17):1098–8. doi: 10.1056/NEJM198710223171717 3657876

[14] Hirooka M Kumagi T Kurose K Nakanishi S Michitaka K Matsuura B. A technique for the measurement of visceral fat by ultrasonography: comparison of measurements by ultrasonography and computed tomography. Intern Med (2005) 44(8):794–9. doi: 10.2169/internalmedicine.44.794 16157975

[15] Sutharsan R O’Callaghan MJ Williams G Najman JM Mamun AA. Rapid growth in early childhood associated with young adult overweight and obesity–evidence from a community based cohort study. J Health Popul Nutr (2015) 33(1). doi: 10.1186/s41043-015-0012-2 PMC5025965 26825961

[16] Kuro-o M. The klotho proteins in health and disease. Nat Rev Nephrol (2019) 15(1):27–44. doi: 10.1038/s41581-018-0078-3 30455427

[17] Flippo KH Jensen-Cody SO Claflin KE Potthoff MJ. FGF21 signaling in glutamatergic neurons is required for weight loss associated with dietary protein dilution. Sci Rep (2020) 10(1). doi: 10.1038/s41598-020-76593-2 PMC7658965 33177640

[18] Maekawa R Seino Y Ogata H Murase M Iida A Hosokawa K. Chronic high-sucrose diet increases fibroblast growth factor 21 production and energy expenditure in mice. J Nutr Biochem (2017) 49:71–9. doi: 10.1016/j.jnutbio.2017.07.010 28886439

[19] Claflin KE Sullivan AI Naber MC Flippo KH Morgan DA Neff TJ. Pharmacological FGF21 signals to glutamatergic neurons to enhance leptin action and lower body weight during obesity. Mol Metab (2022) 64. doi: 10.1016/j.molmet.2022.101564 PMC9403559 35944896

[20] Socha-Banasiak A Michalak A Pacześ K Gaj Z Fendler W Socha A. Klotho and fibroblast growth factors 19 and 21 serum concentrations in children and adolescents with normal body weight and obesity and their associations with metabolic parameters. BMC Pediatr (2020) 20(1). doi: 10.1186/s12887-020-02199-2 PMC7296965 32546231

[21] Gallego-Escuredo JM Gómez-Ambrosi J Catalan V Domingo P Giralt M Frühbeck G. Opposite alterations in FGF21 and FGF19 levels and disturbed expression of the receptor machinery for endocrine FGFs in obese patients. Int J Obes (Lond) (2015) 39(1):121–9. doi: 10.1038/ijo.2014.76 24813368

[22] Rao Z Landry T Li P Bunner W Laing BT Yuan Y. Administration of alpha klotho reduces liver and adipose lipid accumulation in obese mice. Heliyon (2019) 5(4). doi: 10.1016/j.heliyon.2019.e01494 PMC6484204 31049427

[23] Izaguirre M Gil MJ Monreal I Montecucco F Frühbeck G Catalán V. The role and potential therapeutic implications of the fibroblast growth factors in energy balance and type 2 diabetes. Curr Diabetes Rep (2017) 17(6). doi: 10.1007/s11892-017-0866-3 28451950

[24] Landry T Li P Shookster D Jiang Z Li H Laing BT. Centrally circulating α-klotho inversely correlates with human obesity and modulates arcuate cell populations in mice. Mol Metab (2021) 44. doi: 10.1016/j.molmet.2020.101136 PMC7777546 33301986

[25] Behringer V Stevens JMG Deschner T Sonnweber R Hohmann G. Aging and sex affect soluble alpha klotho levels in bonobos and chimpanzees. Front Zool (2018) 15(1). doi: 10.1186/s12983-018-0282-9 PMC6146871 30250491

[26] Arking DE Krebsova A Macek M Macek M Arking A Mian IS. Association of human aging with a functional variant of klotho. Proc Natl Acad Sci U S A (2002) 99(2):856–61. doi: 10.1073/pnas.022484299 PMC117395 11792841

[27] Arking DE Atzmon G Arking A Barzilai N Dietz HC. Association between a functional variant of the KLOTHO gene and high-density lipoprotein cholesterol, blood pressure, stroke, and longevity. Circ Res (2005) 96(4):412–8. doi: 10.1161/01.RES.0000157171.04054.30 15677572

[28] Semba RD Cappola AR Sun K Bandinelli S Dalal M Crasto C. Plasma klotho and cardiovascular disease in adults. J Am Geriatr Soc (2011) 59(9):1596–601. doi: 10.1111/j.1532-5415.2011.03558.x PMC3486641 21883107

[29] Pedersen L Pedersen SM Brasen CL Rasmussen LM. Soluble serum klotho levels in healthy subjects. comparison of two different immunoassays. Clin Biochem (2013) 46(12):1079–83. doi: 10.1016/j.clinbiochem.2013.05.046 23707222

[30] Gkentzi D Efthymiadou A Kritikou D Chrysis D. Fibroblast growth factor 23 and klotho serum levels in healthy children. Bone (2014) 66:8–14. doi: 10.1016/j.bone.2014.05.012 24880094

[31] Sze L Schmid C. Effects of age, sex, and estrogen on serum phosphorus: role for growth hormone and klotho? Am J Kidney Dis (2014) 64(1):157–8. doi: 10.1053/j.ajkd.2014.03.021 24954457

[32] Palmer BF Clegg DJ. The sexual dimorphism of obesity. Mol Cell Endocrinol (2015) 402:113–9. doi: 10.1016/j.mce.2014.11.029 PMC4326001 25578600

[33] Lumish HS O’Reilly M Reilly MP. Sex differences in genomic drivers of adipose distribution and related cardiometabolic disorders: opportunities for precision medicine. Arterioscler Thromb Vasc Biol (2020) 40(1):45–60. doi: 10.1161/ATVBAHA.119.313154 31747800

[34] Araújo J Ramos E Mishra GD Severo M. The use of weight adjusted for height rather than body mass index to assess growth trajectory: results from a population-based cohort. Stat Med (2019) 38(5):855–65. doi: 10.1002/sim.8007 30368858

[35] Corrêa H de L Raab ATO Araújo TM Deus LA Reis AL Honorato FS. A systematic review and meta-analysis demonstrating klotho as an emerging exerkine. Sci Rep (2022) 12(1). doi: 10.1038/s41598-022-22123-1 PMC9585050 36266389

[36] Arroyo E Troutman AD Moorthi RN Avin KG Coggan AR Lim K. Klotho: an emerging factor with ergogenic potential. Front Rehabil Sci (2022) 2. doi: 10.3389/fresc.2021.807123 PMC9397700 36188832

[37] Tan SJ Chu MM Toussaint ND Cai MMX Hewitson TD Holt SG. High-intensity physical exercise increases serum α-klotho levels in healthy volunteers. J Circ Biomarkers (2018) 7. doi: 10.1177/1849454418794582 PMC6100126 30147756

[38] Aczel D Torma F Jokai M McGreevy K Boros A Seki Y. The circulating level of klotho is not dependent upon physical fitness and age-associated methylation increases at the promoter region of the klotho gene. Genes (Basel) (2023) 14(2). doi: 10.3390/genes14020525 PMC9957177 36833453

[39] Jago R Drews KL McMurray RG Thompson D Volpe SL Moe EL. Fatness, fitness, and cardiometabolic risk factors among sixth-grade youth. Med Sci Sports Exerc (2010) 42(8):1502–10. doi: 10.1249/MSS.0b013e3181d322c4 PMC2921216 20139783

[40] Arias Téllez MJ Soto-Sánchez J Weisstaub SG. Physical fitness, cardiometabolic risk and heart rate recovery in Chilean children. Nutr Hosp (2018) 35(1):44–9. doi: 10.20960/nh.1323 29565148

