# Supplementary material for: Higher levels of serum α-Klotho are longitudinally associated with less central obesity in girls experiencing weight gain
Source: Front Endocrinol (Lausanne). 2023 Jul 14;14:1218949. doi: 10.3389/fendo.2023.1218949 (PMC10382686; doi:10.3389/fendo.2023.1218949)
Supplement: Supplementary file 1 [file Table_1.pdf]

Supplementary Material

Supplementary Table S1: Correlations between baseline α-Klotho and the obesity-related parameters studied in all boys and in subgroups according to weight change between baseline and follow-up.

	**Boys (N=101)**	**Weight loss (N=32)**	**Stable weight (N=36)**	**Weight gain (N=33)**
**Baseline**	Baseline α-Klotho (log)	Baseline α-Klotho (log)	Baseline α-Klotho (log)	Baseline α-Klotho (log)
BMI	-0,229*	-0,361*	-0,048	-0,287
BMI SDS	-0,152	-0,292	0,029	-0,206
Waist	-0,181	-0,231	-0,034	-0,292
Waist-to-height ratio	-0,089	-0,081	0,043	-0,261
Body fat mass	-0,308**	-0,473*	-0,184	-0,214
Visc fat	-0,312**	-0,416*	-0,223	-0,356
TG (log)	-0,214*	-0,256	-0,133	-0,265
HOMA-IR	-0,301*	-0,448*	-0,135	-0,176
hsCRP (log)	-0,146	-0,111	-0,040	-0,276
**Follow-up**	Baseline α-Klotho (log)	Baseline α-Klotho (log)	Baseline α-Klotho (log)	Baseline α-Klotho (log)
BMI	-0,136	-0,215	-0,048	-0,263
BMI SDS	-0,073	-0,167	0,028	-0,201
Waist	-0,170	-0,297	-0,061	-0,270
Waist-to-height ratio	-0,084	-0,121	-0,001	-0,223
Body fat mass	-0,041	-0,031	0,055	-0,235
Visc fat	-0,159	-0,111	-0,186	-0,271
TG (log)	-0,142	-0,214	-0,275	0,032
HOMA-IR	-0,175	-0,320	-0,144	-0,182
hsCRP (log)	-0,077	-0,104	0,023	-0,187

Pearson correlation coefficients (r) are shown; *p value <0.05, ** p value <0.01, and ***p value <0.001.
No significant independent associations after correcting for sex, baseline age, baseline puberty status and baseline body surface area (BSA) or weight-for-height ratio were observed.
